# Heart Rate Variability Monitoring during Interferential Current Application in the Lower Back Area: A Cross-Sectional Study

**DOI:** 10.3390/ijerph18073394

**Published:** 2021-03-25

**Authors:** Blanca De-la-Cruz-Torres, Eva Martínez-Jiménez, Emmanuel Navarro-Flores, Patricia Palomo-López, Vanesa Abuín-Porras, Raquel Díaz-Meco-Conde, Daniel López-López, Carlos Romero-Morales

**Affiliations:** 1 Department of Physiotherapy, University of Seville, Avicena Street, 41009 Seville, Spain; bcruz@us.es; 2 Facultad de Fisioterapia y Enfermería, Departamento de Enfermería, Universidad de Castilla la Mancha, 45071 Toledo, Spain; eva.martinez@uclm.es or; 3 Frailty Research Organized Group (FROG), Department of Nursing, Faculty of Nursing and Podiatry, University of Valencia, 46001 Valencia, Spain; emmanuel.navarro@uv.es; 4 University Center of Plasencia, Faculty of Podiatry, Universidad de Extremadura, 10600 Badajoz, Spain; patibiom@unex.es; 5 Faculty of Sport Sciences, Universidad Europea de Madrid, Villaviciosa de Odón, 28670 Madrid, Spain; raquel.diazmeco@universidadeuropea.es (R.D.-M.-C.); carlos.romero@universidadeuropea.es (C.R.-M.); 6 Research, Health and Podiatry Group, Department of Health Sciences, Research, Faculty of Nursing and Podiatry, Universidade da Coruña, 15403 Ferrol, Spain; daniellopez@udc.es

**Keywords:** interferential current, heart-rate variability, autonomic balance, sensor technology

## Abstract

Vasovagal reactions may occur occasionally during electrical stimulation using interferential current (IFC). The purpose of this study was to examine variations in autonomic activity during the application of IFC in asymptomatic participants by analysis of their heart rate variability (HRV). Seventy-three male volunteers were randomly assigned to a placebo group (*n* = 36; HRV was documented for 10 min, both at rest and during a placebo intervention) and an intervention group (*n* = 37; HRV was documented for 10 min in two conditions labelled as (1) rest and (2) application of IFC technique on the lumbar segment). The diameters of the Poincaré plot (SD1, SD2), stress score (SS), and the ratio between sympathetic and parasympathetic activity (S/PS) were measured. After interventions, differences amongst the placebo group and the IFC group were found in SD2 (*p* < 0.001), SS (*p* = 0.01) and S/PS ratio (*p* = 0.003). The IFC technique was associated with increased parasympathetic modulation, which could induce a vasovagal reaction. Monitorization of adverse reactions should be implemented during the application of IFC technique. HRV indicators might have a part in prevention of vasovagal reactions. Further studies in patients with lumbar pain are needed to explore possible differences in HRV responses due to the presence of chronic pain.

## 1. Introduction

The definition of interferential current (IFC) therapy involves the therapeutic application of a method of transcutaneous electrical stimulus through medium frequency current [1,2]. In this technique, an amplitude-modulated low frequency current is obtained through the interaction of two medium-frequency currents with minor differences between them. The clinical relevance of this modality of electrical stimulation lays in the fact that it has low impedance regarding the human skin, therefore, this frequency has the capacity of reaching deeper in the soft tissues of the musculoskeletal system. In order to penetrate this far, historically used treatments such as direct current or low frequency alternating currents would need a large intensity that would make the treatment painful for the patient. IFC therapy has been, consequently, studied in the treatment of deep tissue conditions, especially those concerning pain management [3,4].

Increased blood flow and modification of the peripheral circulation have been reported as physiological effects of electrical stimulation [5]. Different physiological responses can be caused by appropriated dosing of electrical current application [6]. Thus, the influence of electrical stimulation over the autonomic nervous system needs to be addressed in order to establish accurate protocols to prevent potential adverse reactions of importance, such as vasovagal syncope.

As in other physiotherapeutic procedures [7,8,9], mild vagal reactions are common during IFC therapy in clinical practice. Moreover, visceral effects (e.g., sickness, vomiting, dilated pupils) may appear, particularly in association with stronger stimulation and/or longer retention time. Sporadically, vasovagal reactions may occur, which are clinical events with vagus nerve implication that are associated with dizziness and sickness sensations. Rarely, vasovagal responses may have as an outcome vasovagal syncope. Hee-Kyung et al. [10] investigated the changes in the autonomic nervous system before, immediately after, and 30 min after IFC technique using doppler ultrasonography, measuring blood flow to determine blood speed and vessel size. Their results showed disbalances in the sympathetic nervous system.

Heart rate variability (HRV) has been proven valuable to measure discordances between sympathetic and parasympathetic activity [11]. HRV has been studied by several authors using various conditions that have validated it as a reliable instrument to evaluate and to assess the state of sympathetic and parasympathetic components of the autonomous nervous system [12,13,14]. In this study, HRV was used as a marker to detect autonomic disbalance characterized by a preponderance of parasympathetic activity that can precede vasovagal responses.

The purpose of this study was to evaluate the variations in sympathetic and parasympathetic activity (using HRV measures) during IFC therapy measured on the lumbar segment on asymptomatic volunteers, in order to help to further the understanding of the common vagal reactions observed in clinical practice during the application of this technique. 

## 2. Materials and Methods

**Figure 1:**
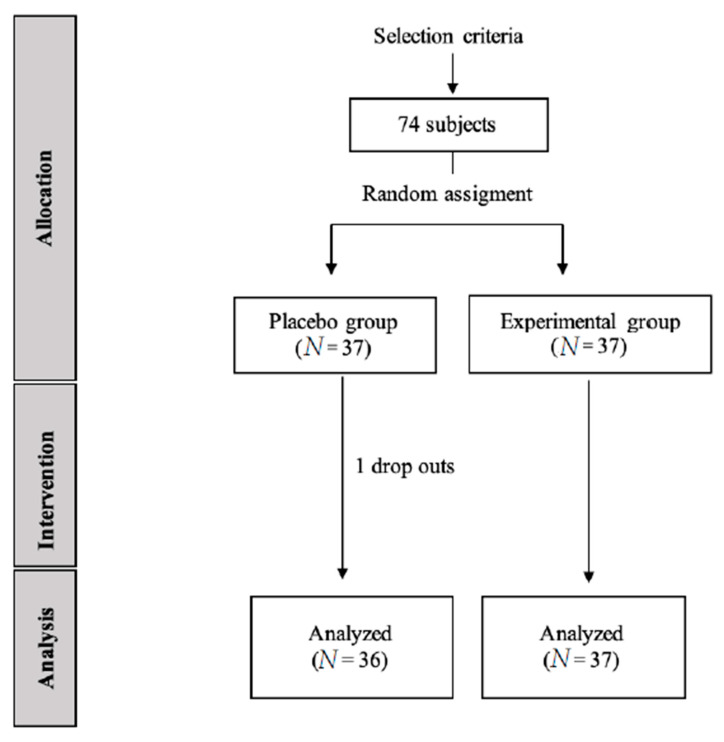
CONSORT flow diagram of subject recruitment.

Seventy-three asymptomatic adult volunteers (51 female, 22 male) were enrolled, recruited from a private clinic, and split randomly into two groups: (1) a placebo group (*n* = 36) and (2) an experimental group (*n* = 37). Exclusion criteria were: (a) any uncontrolled neurological or cardiac disorder; (b) personal psychological apprehension scale (PPAS) score higher than 37.5 [15]; (c) contraindication for electrical stimulation; and (d) any sensory disorder (Figure 1).

### 2.1. Sample Size

G*Power software was employed for the sample size calculation by the difference between the placebo group and experimental group using the transverse axis of Poincaré plot variable of a pilot study (*n* = 10) divided in two groups (mean ± SD), 5 subjects from the control group (32.38 ± 6.13) and 5 subjects for the intervention group (36.29 ± 7.30). For the sample size calculation, a power of 0.80, an α error of 0.05, and effect size of 0.6 with one-tailed hypothesis were employed. In conclusion, a sample of 74 individuals was included. However, from the placebo group, one individual was discarded because he presented discomfort in the baseline assessment.

### 2.2. Ethics

The local ethics committee (University Hospital Virgen Macarena-Virgen del Rocio; code 01/2017) approved the study according to the Declaration of Helsinki statements. All subjects signed written informed consent to enroll in the study. This study was registered in the clinical trials database (clinicaltrials.gov, accessed on 28 March 2018), with registry number NCT03483064.

### 2.3. Procedure

**Figure 2:**
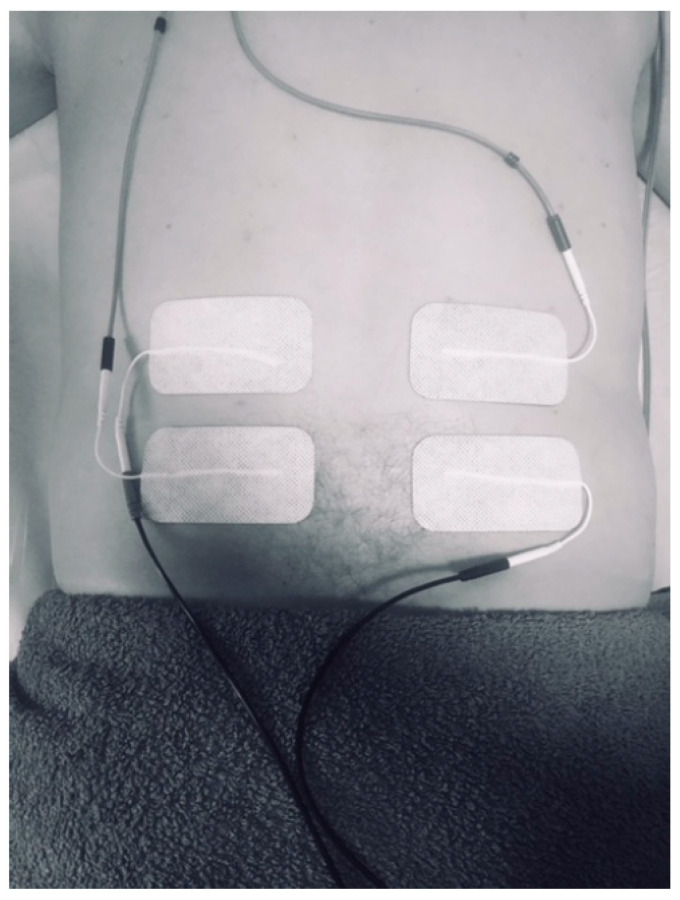
Position of electrodes during application of the Interferential Current technique. Subjects were asked to lay down in a prone position, with the low back area unclothed.

First, all subjects completed the PPAS, in order to assess the psychological apprehension of the subjects regarding electro physiotherapeutic intervention [15]. All patients were also asked to report any adverse events that they experienced during the research. No adverse events were reported. Participants were randomly assigned to either placebo or intervention group by means of sealed, opaque envelopes containing the allocation of the patient previously determined by a computer-generated, randomized table of numbers. The information on these envelopes was delivered to the physiotherapist in charge of the intervention, who was blinded to the baseline assessment. Once the allocation of the participant was determined, HRV was recorded with participants laying in the prone position. Participants were asked to fast overnight, and the testing was conducted early in the morning. HRV was recorded for the placebo group (CG) for 10 min, both at rest and during placebo intervention in the lower back, with the electrodes placed but without application of electrical current. Both group were instructed in the line that they may or may not have any sensation during the application of the IFC, in order to guarantee that the individuals were blinded to their allocation. HRV was recorded for the experimental group (EG) for 10 min, in the same conditions as the placebo group. Although all study participants were asymptomatic, the lower back was selected as the intervention area, since lumbar pain is a common indication for IFC therapy [16,17]. As previous studies have described [18], IFC therapy consisted in the application of a transregional interferential current in a quadripolar technique with a carrier frequency of 4000 Hz, an amplitude-modulated frequency of 100 Hz. The model used was certified and developed for medical purposes (Sonopuls 692^®^, Enraf-Nonius BV, Rotterdam, The Netherlands) [19]. This model is commonly used in clinical practice, although, to the authors’ knowledge, it lacks a validation study up to this date. Participants were requested to lay prone and unclothe their lumbar area. Four self-adhesive electrodes (6 × 8 cm) (Pals Platinum© type, Axelgaard Manufacturing Co. Ltd., Fallbrook, CA, USA) were placed at the level of the first and fifth lumbar vertebrae using a crossed pattern (Figure 2): the electric current in each channel crossed the area of intervention (one electrode was located on the right of L1 and the other on the left of L5 for the first channel, whereas one electrode was located on the left of L1 and the other on the right of L5 for the second channel) (Figure 2). Following previous protocols involving IFC in the lumbar area, the intensity of the current was adapted to the participants’ tolerance, trying to produce a “pins-and-needles” sensation, without a visible muscle contraction [20]. The intervention was administrated by an experienced physiotherapist. 

**Figure 3:**
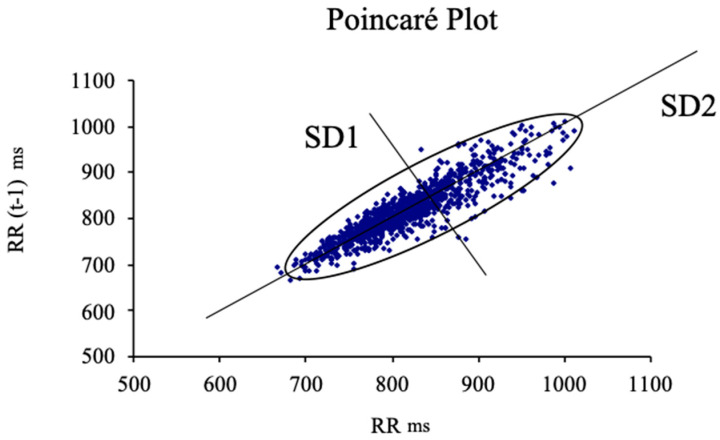
Model of Pointcaré plot.

The procedure for the HRV assessment was carried on using a heart rate (HR) monitoring device (Firstbeat Bodyguard, Firstbeat Technoogies, Iyväskylä, Finland) [21]. For the download and importation of the data involving the RRI series, an uploader software (Firstbeat, Firstbeat Technologies) and the software package Kubios (University of Eastern Finland, Kuopio, Finland) were used. Regarding the calculation of the disbalance between sympathetic and parasympathetic activity, an HRV scheme based on the Poincaré plot was used, which reflects HRV fluctuations and may be adjusted to an ellipse to interpret their diameters [22,23]. The Poincaré dispersion graphic (Figure 3) is defined as a graphic representation of the dynamic behavior of the heart signal, showing visually the variations on the RR time series, usually adopting an elliptical shape [22,23]. The transverse axis (SD1) reflects activity of the parasympathetic system [24] and the longitudinal axis was used for the sympathetic activity assessment, even though its physiological meaning is controversial [20].

Following studies by Naranjo et al. [25], two more indicators were given consideration, the stress score (SS) and the sympathetic-parasympathetic ratio (S-PS). The first one is defined as “the inverse of the diameter SD2 multiplied by 1000” (SS = 1000 × 1/SD2) and is defined as directly proportional to sympathetic activity located in the sinus node. The S/PS ratio is expressed as “the quotient of SS and SD1” (S/PS ratio = SS/SD1) and defined by Naranjo et al. [25] as an indicator of autonomic balance, considering it a sign of the ratio amongst sympathetic and parasympathetic activity.

### 2.4. Statistical Analysis

For statistical analysis, the Kolmogorov–Smirnov test was used to assess the normality of the data. Additionally, a 95% CI was stablished for all the variables, which were expressed in terms of mean and standard deviation. Besides, the data analysis was performed by a mixed model analysis of variance ANOVAs (2 × 2) with one between-group factor (placebo group versus intervention group) and one within-group factor (baseline versus IFC). The intraclass correlation coefficient (ICC) was used to determine the reliability of each measurement. Coefficients <0.20 indicate slight agreement, between 0.20 and 0.40 indicate fair reliability, between 0.41 and 0.60 indicate moderate reliability, between 0.61 and 0.80 indicate substantial reliability, and between 0.81 and 1.00 indicate almost perfect reliability. Effect sizes (ES) were also calculated using Cohen’s d coefficient. A small difference is set by an effect size between ≥0.2 and <0.5, moderate is placed between ≥0.5 and <0.8, and it is considered large if it is ≥0.8 [26]. Statistical Package for the Social Sciences (SPSS) v.21 (SPSS Inc., Chicago, IL, USA) was used for the data analysis with an α error of 0.05 (95% confidence interval) and a desired power of 80% (β error of 0.2) used for all statistical tests.

## 3. Results

Demographic variables did not show any significant baseline differences between the placebo and experimental groups, such as age (20.53 ± 2.83 vs. 22.22 ± 3.57 years, *p* = 0.16), weight (70.89 ± 7.73 vs. 75.76 ± 11.11 kg, *p* = 0.05), height (177.44 ± 5.41 vs. 178.97 ± 7.69 cm, *p* = 0.33), and body mass index (22.47 ± 1.84 vs. 23.65 ± 3.22, *p* = 0.06). The scores for the PPAS scale were 23.97 ± 5.17 for the placebo and 23.51 ± 4.15 for the experimental group (*p* = 0.14) (Table 1).

Table 2 shows the baseline, post-intervention scores, and the mean differences of the between-groups and within-group comparison for the HRV parameters. There were no differences between the placebo group and the IFC group in the baseline measurements (all *p* > 0.05); however, after the interventions, the groups displayed significantly different results. Compared with the baseline values, the placebo group only exhibited a statistically significant increase in SD1 after intervention (34.36 ± 11.42 vs. 37.49 ± 13.12, *p* = 0.03, d = 0.2). Compared with baseline measures, the IFC group showed statistically significant increases in SD1 (31.57 ± 12.73 ms vs. 42.13 ± 9.78 ms, *p* < 0.001, d = 0.9) and SD2 (76.82 ± 23.84 ms vs. 103.68 ± 29.61 ms, *p* < 0.001, d = 1.0), and statistically significant decreases in SS (14.62 ± 4.56 ms vs. 11.74 ± 3.95, *p* < 0.001, d = 0.7) and S/PS ratio (0.60 ± 0.43 vs. 0.34 ± 0.18, *p* < 0.001, d = 0.8) after intervention. The ICC was 0.83 (0.74–0.90) for the SD1, 0.47 (0.15–0.66) for the SD2, 0.73 (0.56–0.83) for SS, and 0.72 (0.55–0.83) for the S/PS ratio.

After the interventions, differences were found between the placebo group and the IFC group in SD2 (89.41 ± 17.73 ms vs. 103.68 ± 29.61 ms, *p* < 0.05, d = 0.6), SS (14.06 ± 5.31 vs. 11.74 ± 3.95, *p* < 0.05, d = 0.6), and S/PS ratio 0.53 ± 0.41 vs. 0.34 ± 0.18, *p* < 0.05, d = 0.6). Figure 3 shows the comparison of SD1, SS, and S/PS ratio between baseline and during intervention for both groups.

**Figure 4:**
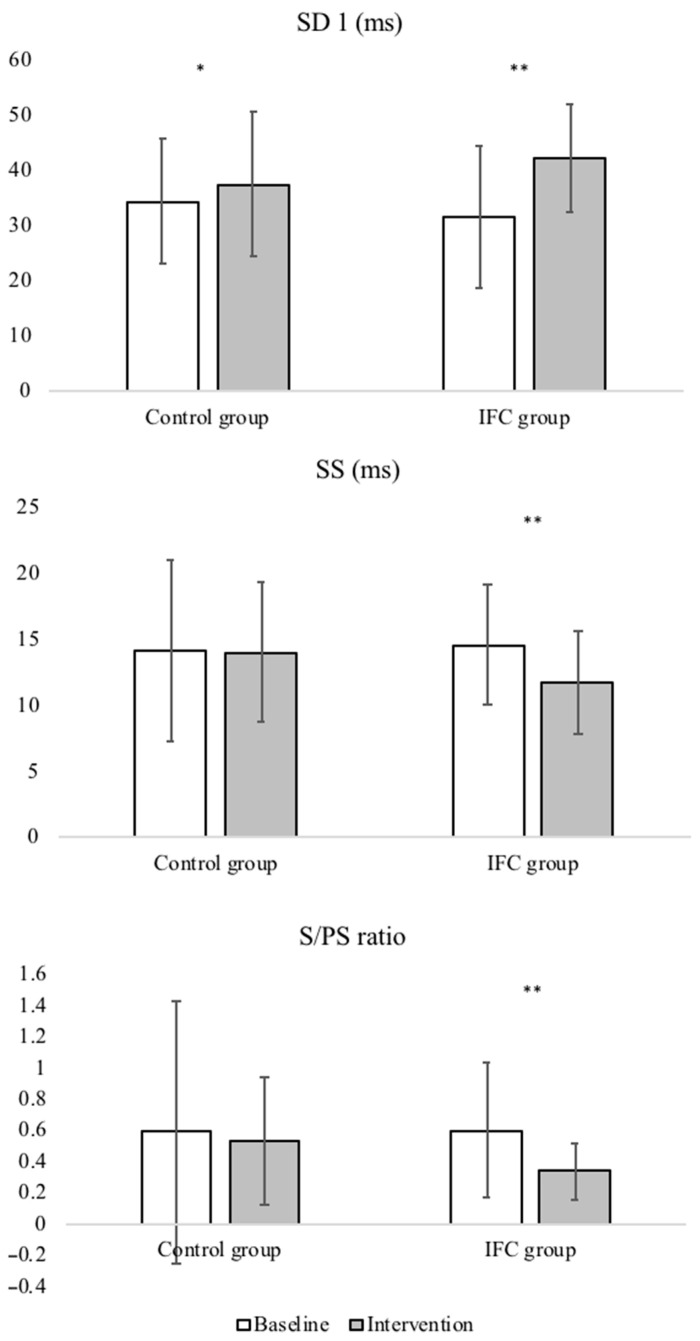
Comparison of SD1, SS, and S/PS ratio between baseline and during intervention for both groups. * Indicates statistically significant within-group differences (*p* < 0.05); ** Indicates statistically significant within-group differences (*p* < 0.001).

Interventions in the control and IFC groups consisted of IFC intervention without and with current, respectively. SD1 = transverse axis of Poincaré plot; SD2 = longitudinal axis of Poincaré plot, SS = stress score (inverse of diameter SD2 × 1000); S/PS ratio = quotient of SS and SD1. Figure 4 shows the comparison of SD1, SS, and S/PS ratio between baseline and during intervention for both groups.

## 4. Discussion

The aim of this study was not to evaluate the therapeutic technique; instead, we intended to explore whether vagal reactions could appear during its use, detection of which may be important to ensure safe clinical practice. The core result of this study was the presence of a substantial autonomic disbalance, that could lead to a hypothetical vasovagal response, during application of IFC therapy in asymptomatic subjects, measured by HRV. These findings may be relevant for clinical practice as significant vasovagal reactions (including syncope) during the application of IFC therapy could potentially be avoided. Consequently, it is important to recommend physiotherapists to take precautions when performing this technique, and to be ready to tend to any adverse reactions. 

A statistically significant increase was reported in the marker of parasympathetic activity, SD1; additionally, a statistically significant decrease in SS was only observed in the IFC group. Thus, this suggests that application of the IFC therapy causes an autonomic disbalance, which can affect the balance between parasympathetic and sympathetic activity, and is characterized by a predominance of parasympathetic activity, reflected as S/PS ratio (Table 2).

These results show that the application of IFC therapy was implicated in the vagal response. Nevertheless, these outcomes imply a positive consequence, since this technique results in a central nervous system activation in the form of a segmentary response that switches on the restoration processes. This segmentary response is recognized as the response of the organism to a threat, also known as the fight–flight response [27,28].

According to previous studies [7,8,14], it appears that this autonomic disbalance was elicited by IFC therapy, with no relation with the participants’ attitudes towards the therapy, which was mostly confident. In this study, the possibility of the variation of the HRV hypothetically caused by the perception of the current was controlled by two strategies. First, every subject filled in the PPAA questionnaire to ensure that they did not have a possible negative attitude to the treatment with currents; and in second place, the placebo subjects were led to believe that they were also being treated with currents, although they did not notice it since the device was turned off. Moreover, participants in both the placebo and IFC groups recorded a mean of 23 points on the PPAS, a value substantially below the 37.5 score that is labelled as “uneasiness” [15]. 

The examination of HRV has been present in studies involving some pathologies [13,28,29] and sports performances [12,30]. HRV is now being explored as a valid instrument to evaluate the physiological effect of different physiotherapeutic techniques. Some authors explored HRV in the context of therapeutic massage [31,32], craniosacral therapy [33], acupuncture [26,34], and percutaneous needle electrolysis (PNE) [35,36]. Moreover, other investigations have included certain practices to reduce physiological stress, based on their reports of an increase in HRV (increased parasympathetic activity) during the application of various techniques [31,33,37]. Several studies addressing autonomic system activity during acupuncture technique treatment reported increased parasympathetic activity [34,38]. Meanwhile, other authors have also observed larger parasympathetic activity during the application PNE technique [35,36]. In this study, using two groups of asymptomatic subjects (placebo group and IFC group), we observed only an autonomic disbalance towards vagal dominance in the IFC group, due mainly to the decrease in sympathetic activity (SS) (Table 2). Therefore, when a physiotherapist uses IFC therapy, it is possible that subjects experience an autonomic disbalance. Accordingly, clinicians should expect and prevent the potential apparition of adverse responses (vasovagal syncope). Although none of the expected responses can be considered as “dangerous”, it is important for the clinician to be able to explain the reasons for their patients’ uneasiness (if present) and to create a safe environment in case of vasovagal syncope to prevent falls.

### Limitations

IFC therapy can result in an autonomic disbalance; therefore, the technique has a local and a segmental effect, which was not investigated in the current study. Thus, future studies could aim to clarify the therapeutic mechanisms regarding local and segmental responses during the application of IFC. In this study, adiposity was not controlled, so its possible interference with the IFC is yet to be explored in further studies. Additionally, the current study was performed in asymptomatic subjects. However, over time, HRV can be decreased by the effect of constant, chronic pain, which activates the individual stress response [39]. For this reason, we think that it would be pertinent to perform this study in patients with lumbar pain to assess this population’s particular response to IFC technique.

## 5. Conclusions

In conclusion, an increase in parasympathetic activity assessed with HRV was observed during the application of the IFC technique. Regarding this, during clinical practice, indicators of adverse vasovagal reactions should be monitored when applying this therapy. The use of HRV indicators could potentially be valuable as an indicator for primary detection of adverse vasovagal reactions during application of the IFC. Further studies in patients with lumbar pain are needed in order to explore possible differences in their responses due to the presence of chronic pain.

## Figures and Tables

**Table 1:** Baseline clinical and demographic features of the sample.

	Total Sample (*N* = 73)	IFC Group (*n* = 37)	Control Group (*n* = 36)	*p*-Value *
Mean age (years)	21 (5.12)	22 (3.57)	20 (2.83)	0.16
Height (cm)	178.22 (6.66)	178.97 (7.69)	177.44 (5.4)	0.33
Body Mass (Kg)	73.36 (9.84)	75.76 (11.11)	70.89 (7.73)	0.07
Body Mass Index (Kg/m^2^)	23.06 (2.68)	23.64 (3.22)	22.47 (1.83)	0.16
Gender (F/M)	73 (51/22)	37 (25/12)	36 (26/10)	n/a
PPAS	24.75 (4.80)	23.51 (4.15)	23.97 (5.17)	0.14

Data are reported as mean (SD). PPAS, Personal Psychological Apprehension Scale. * Between-groups statistical significance (one-factor ANOVA) n/a not applicable.

**Table 2:** Baseline, post-intervention, and mean score changes in heart rate variability parameters.

Variable	Group	Baseline	Intervention	Within-Group Mean Changes	Between-Groups Mean Changes
SD1 (ms)	Control group	34.36 (30.49/38.22)	37.49 (30.49/38.22)	3.13 (5.97/0.29) *	4.63 (0.75/10.02)
	IFC group	31.57 (27.32/35.81)	42.13 (33.05/41.93)	10.56 (13.24/7.88) **	
SD2 (ms)	Control group	82.96 (72.71/93.21)	89.41 (83.41/95.41)	6.45 (0.35/13.26)	14.26 (2.83/25.7) †
	IFC group	76.82 (68.88/84.76)	103.68 (100.48/106.88)	26.86 (16.9/36.82) **	
SS (ms)	Control group	14.13 (11.79/16.48)	14.46 (12.67/16.26)	0.33 (1.98/1.32)	2.72 (0.54/4.90) †
	IFC group	14.62 (13.10/16.14)	11.74 (10.42/13.06)	2.87 (1.36/4.38) **	
Ratio S/PS	Control group	0.59 (0.31/0.88)	0.47 (0.32/0.62)	0.06 (−0.12/0.25)	0.19 (0.04/0.34) †
	IFC group	0.60 (0.46/0.75)	0.90 (0.67/01.14)	0.26 (0.13/0.39) **	

Data are reported as mean (95% confidence level). Between-groups statistical significance (ANOVA 2 × 2). * Indicates statistically significant within-group differences (*p* < 0.05), ** Indicates statistically significant within-group differences (*p* < 0.001), † Indicates statistically significant between-group differences (*p* < 0.05).

## Data Availability

Data available from the authors under request.
